# Supplementary material for: Distinct tumor genomic signatures underlie canine macrophage polarization
Source: PLoS One. 2026 Apr 24;21(4):e0346239. doi: 10.1371/journal.pone.0346239 (PMC13108725; doi:10.1371/journal.pone.0346239)
Supplement: S1 File — Full-membrane chemiluminescent images captured on a BioRad ChemiDoc system for CCL3 (~10 kDa, top panels) and α-tubulin loading control (~50 kDa, bottom panels). Left panels show standard chemiluminescence exposures; right panels show merged chemiluminescence and colorimetric membrane images. Lanes are labeled as follows: L, molecular weight ladder; lanes 1–5, Nike cells (not used in analysis, marked with X); lanes 6–10, DH82 cells. Treatment conditions were: 100 nM (lanes 1, 6), 50 nM (lanes 2, 7), and 10 nM (lanes 3, 8) CCL3-targeting siRNA; non-targeting control siRNA (lanes 4, 9); and wild-type untreated cells (lanes 5, 10). Cell lysates were collected 72 h post-transfection. Cropped regions from lanes 6–10 were used for densitometric quantification in S3 Fig. (PDF) [file pone.0346239.s001.pdf]

L= ladder
1-5: Nike (not used)
6-10: DH82
1,6: 100 nM siRNA
2,7: 50 nM siRNA
3,8: 10 nM siRNA
4,9: Non-targeting control siRNA
5,10: Wild-type cells

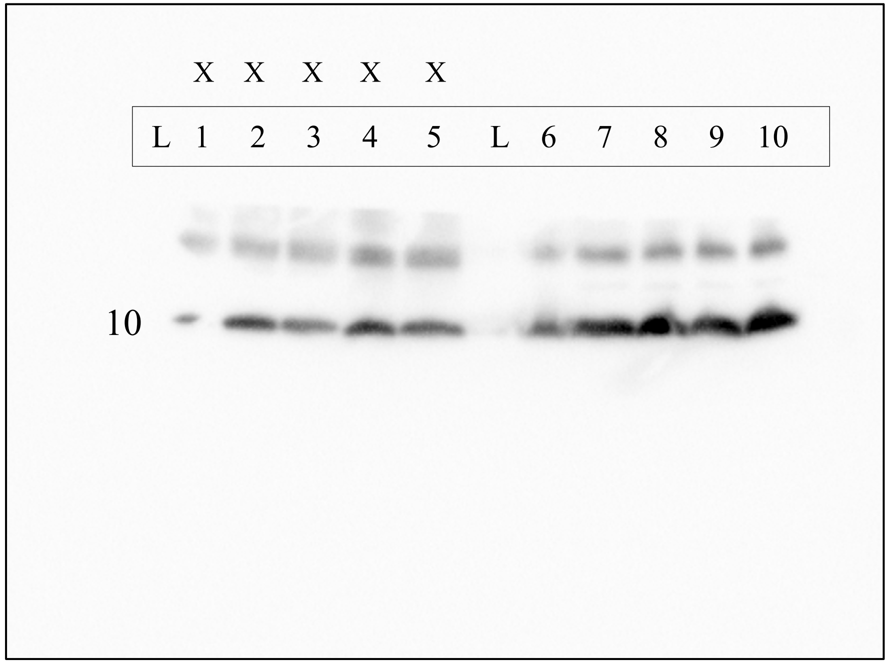

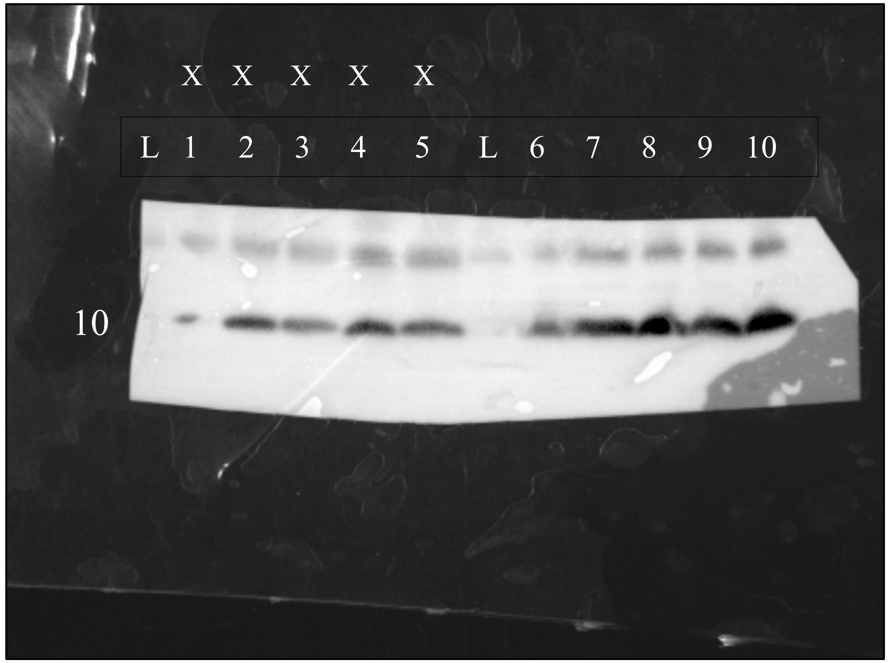

CCL3 (~10 kDa)

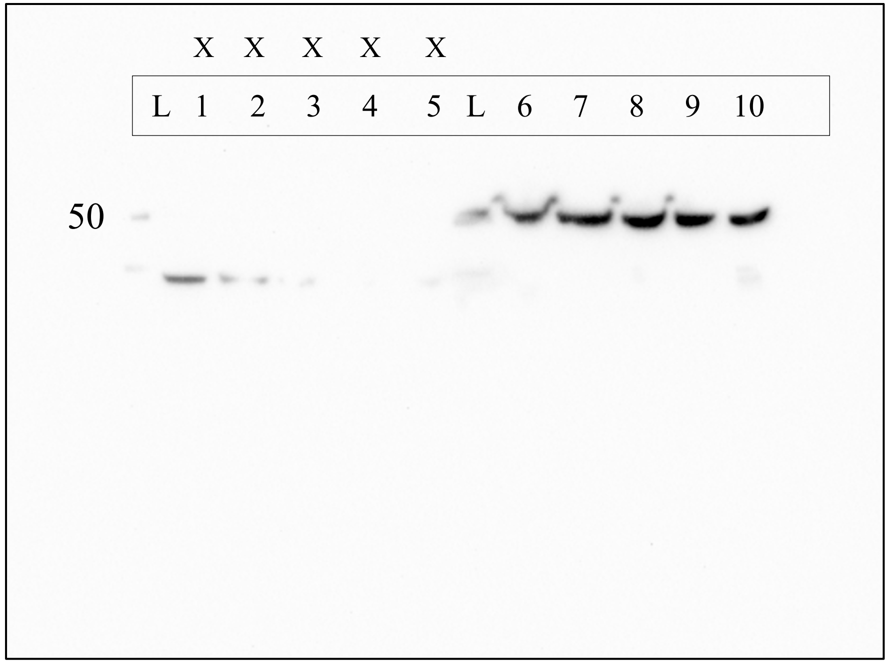

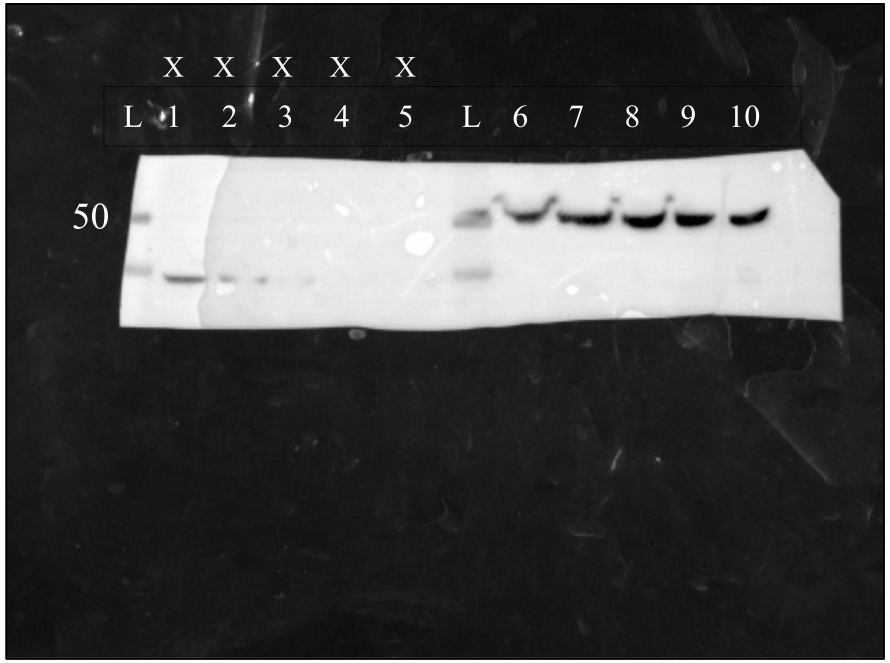

α-tubulin (~50 kDa)

Chemiluminescence, BioRad ChemiDoc
