# Supplementary figures and images for: Distinct tumor genomic signatures underlie canine macrophage polarization
Source: PLoS One. 2026 Apr 24;21(4):e0346239. doi: 10.1371/journal.pone.0346239 (PMC13108725; doi:10.1371/journal.pone.0346239)

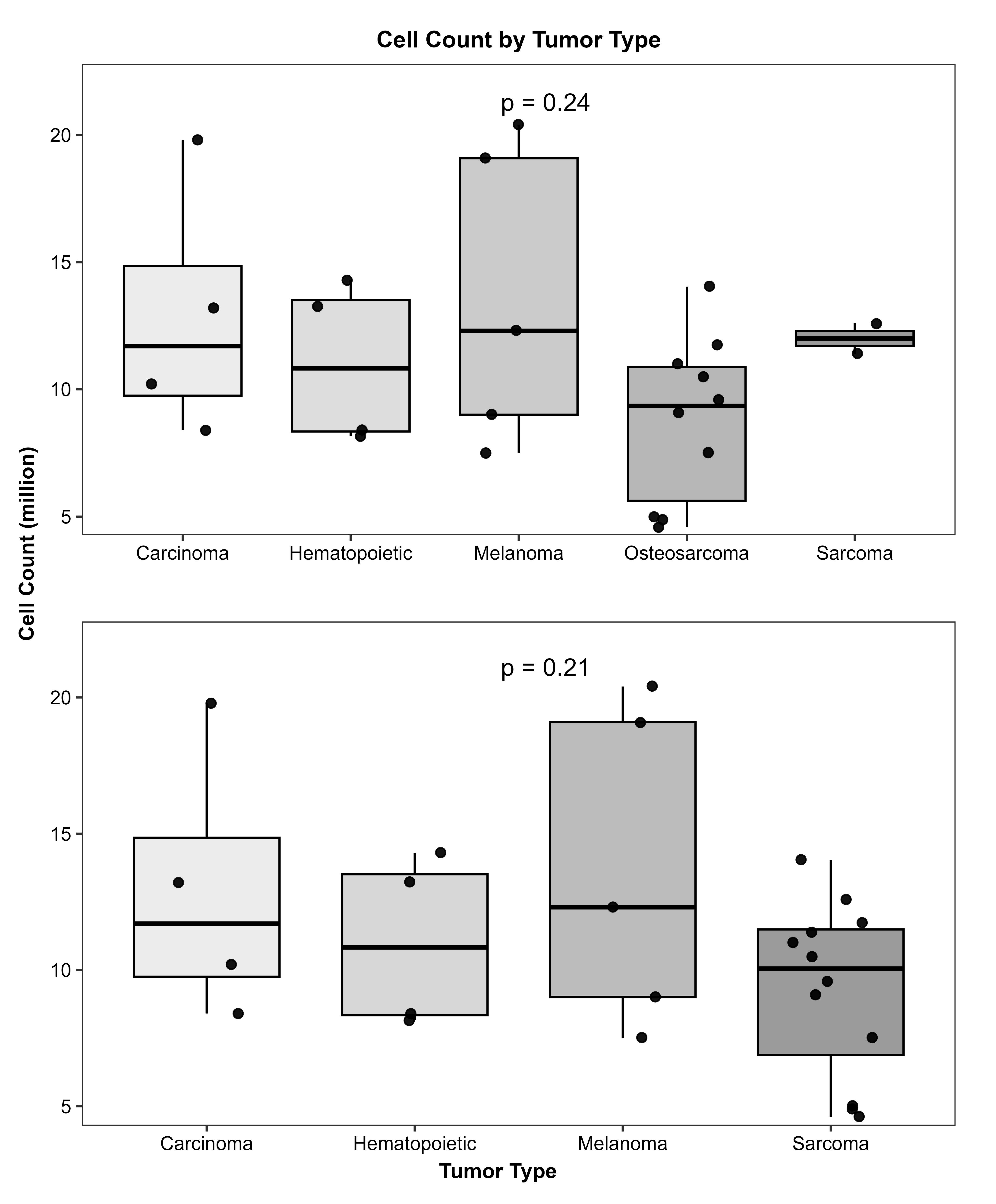

Supplement: S1 Fig — Top: five-category grouping with osteosarcoma shown separately from other sarcomas. Bottom: four-category grouping with osteosarcoma grouped with other sarcomas. No differences were detected between groups (one-way analysis of variance: five-category F(4,20) = 1.488, p = 0.243; four-category F(3,21) = 1.645, p = 0.209). Data met ANOVA assumptions. Points represent individual donor measurements; box plots show median and quartiles. (TIFF) [file pone.0346239.s002.tiff]

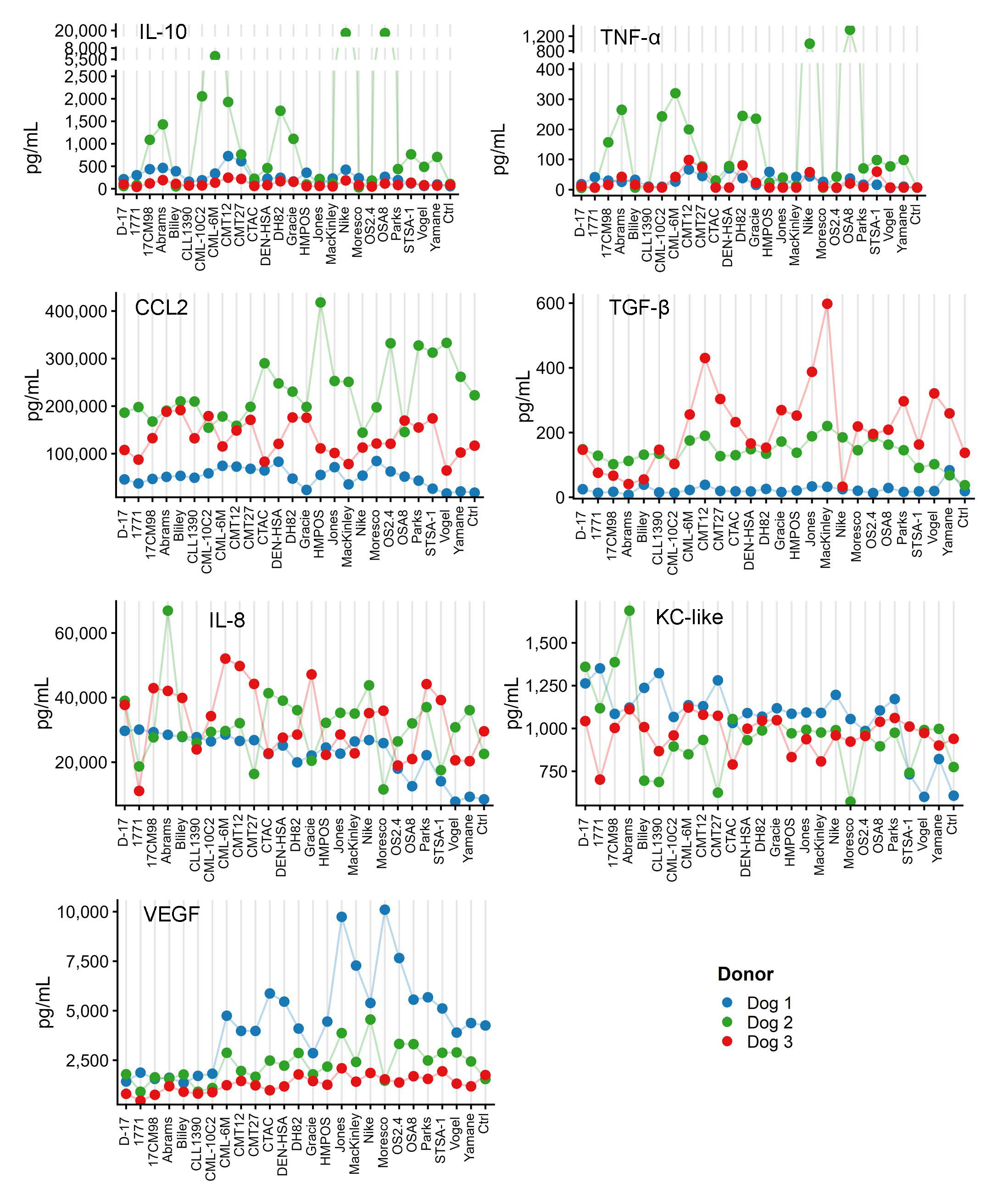

Supplement: S2 Fig — (TIF) [file pone.0346239.s003.tif]

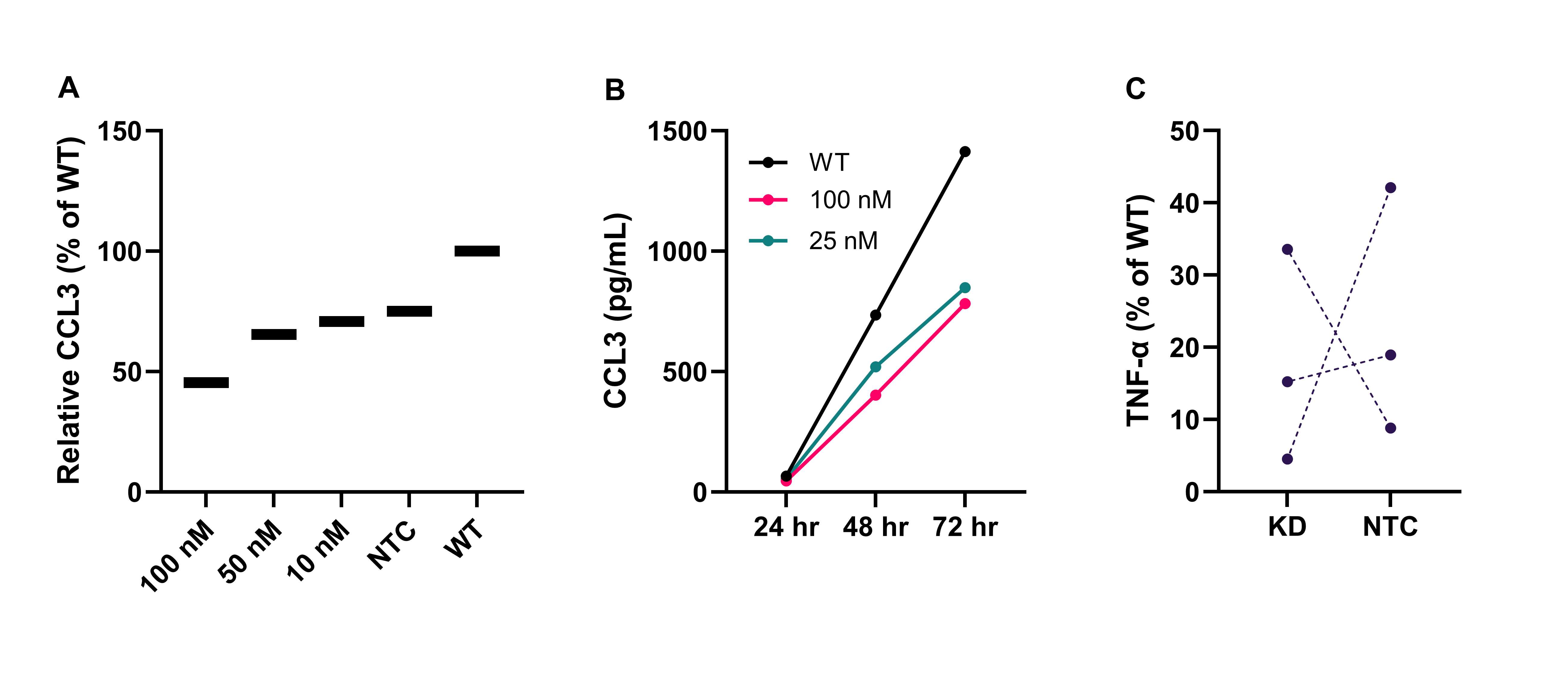

Supplement: S3 Fig — (A) Densitometric quantification of western blot bands from DH82 cell lysates collected 72 h after transfection with increasing concentrations of CCL3-targeted siRNA (10–100 nM), non-targeting control (NTC) siRNA, or untreated wild-type (WT) cells. Band intensities were normalized to WT levels. (B) Absolute CCL3 concentrations measured by ELISA in culture supernatants from WT, 25 nM, and 100 nM siRNA-treated DH82 cells at 24, 48, and 72 h post-transfection. (C) TNF-α secretion from three canine donor macrophages following 24 h exposure and 24 h washout to tumor-conditioned medium (TCM) from WT, NTC, or CCL3-KD (KD) DH82 cells. TNF-α values for each donor were normalized to the WT condition (set to 1). Both KD- and NTC-derived TCM induced significantly lower TNF-α secretion than WT (one-sample t-test vs 1, p = 0.0106 and 0.0159 respectively), whereas KD did not differ significantly from NTC when analyzed by either a one-sample t-test normalized to NTC = 1 or by a paired two-sample t-test (p > 0.05). Color-coded by donor. (TIF) [file pone.0346239.s004.tif]
