# Supplementary material for: Distinct tumor genomic signatures underlie canine macrophage polarization
Source: PLoS One. 2026 Apr 24;21(4):e0346239. doi: 10.1371/journal.pone.0346239 (PMC13108725; doi:10.1371/journal.pone.0346239)
Supplement: S1 Table — Asterisks denote cell lines used only in the validation study. (DOCX) [file pone.0346239.s005.docx]

**S1 Table****: Canine cancer cell lines used.**

| **Cell Line** | **RRID** | **Tumor type** |
| --- | --- | --- |
| 1771 | CVCL_0B18 | Lymphoma |
| 17CM98 | CVCL_0C82 | Melanoma |
| Abrams | CVCL_L309 | Osteosarcoma |
| Bliley | CVCL_DN27 | Urothelial carcinoma |
| CLL-1390 | CVCL_DI61 | Leukemia |
| CML-10C2 | CVCL_0D25 | Melanoma |
| CML-6M | CVCL_0D24 | Melanoma |
| CMT12 | CVCL_L329 | Mammary carcinoma |
| CMT27 | CVCL_1R41 | Mammary carcinoma |
| CTAC | CVCL_4155 | Thyroid adenocarcinoma |
| D-17 | CVCL_1916 | Osteosarcoma |
| DEN | CVCL_1R33 | Hemangiosarcoma |
| DH82 | CVCL_2018 | Histiocytic sarcoma |
| Gracie | CVCL_L353 | Osteosarcoma |
| HMPOS | CVCL_L355 | Osteosarcoma |
| Jones | CVCL_DN28 | Melanoma |
| MacKinley | CVCL_L362 | Osteosarcoma |
| Nike | CVCL_DN29 | Histiocytic sarcoma |
| Moresco | CVCL_L363 | Osteosarcoma |
| OS2.4 | CVCL_L370 | Osteosarcoma |
| OSA8 | CVCL_L405 | Osteosarcoma |
| Parks | CVCL_DN32 | Melanoma |
| STSA-1 | CVCL_D274 | Soft tissue sarcoma |
| Vogel | CVCL_L422 | Osteosarcoma |
| Yamane | CVCL_DN30 | Osteosarcoma |
| Angus* | CVCL_WS61 | Urothelial carcinoma |
| Tyler1* | CVCL_WS64 | Urothelial carcinoma |
| SB* | CVCL_5I33 | Hemangiosarcoma |
| CIN* | CVCL_WS63 | Hemangiosarcoma |

Asterisks (*) denote cell lines used only in the validation study.
