# Supplementary material for: Distinct tumor genomic signatures underlie canine macrophage polarization
Source: PLoS One. 2026 Apr 24;21(4):e0346239. doi: 10.1371/journal.pone.0346239 (PMC13108725; doi:10.1371/journal.pone.0346239)
Supplement: S2 Table — MC = male castrated, MI = male intact, OSA = osteosarcoma, L = low (below reference range). (DOCX) [file pone.0346239.s006.docx]

**S2 Table:** **Donor characteristics for initial tumor-conditioned media generation.**

| **Breed** | **Age (y)** | **Sex** | **Weight (kg)** | **Diagnosis** | **Monocyte Count (K/µL)** |
| --- | --- | --- | --- | --- | --- |
| Mastiff | 8.8 | MC | 74.3 | Periosteal OSA | 0.1 (L) |
| Great Dane | 1.2 | MI | 52.6 | Metastatic OSA | 0.4 |
| Goldendoodle | 7.1 | MC | 32.7 | Multicentric T cell lymphoma | 0.9 |

^a^MC = male castrated, MI = male intact, OSA = osteosarcoma, L = low (below reference range)
