# Supplementary material for: Distinct tumor genomic signatures underlie canine macrophage polarization
Source: PLoS One. 2026 Apr 24;21(4):e0346239. doi: 10.1371/journal.pone.0346239 (PMC13108725; doi:10.1371/journal.pone.0346239)
Supplement: S6 Table — Cell lines were ranked by mean modified z-scores and divided into top and bottom quartiles for each cytokine (n = 6 per group for most cytokines). Statistical tests confirm significant differences in cytokine stimulation between groups. Test selection based on normality: Welch's t-test or Student's t-test for normally distributed data, Mann-Whitney U test otherwise. These quartile groups were then used for DESeq2 differential expression analysis. (DOCX) [file pone.0346239.s010.docx]

**S6 Table:** **Top and bottom quartiles used for DEG analysis*.***

| **Cytokine** | **Top Stimulators** | **Bottom Stimulators** | **Test** | ***p*-value** |
| --- | --- | --- | --- | --- |
| VEGF | Jones  Moresco  OS2.4  Nike  MacKinley  OSA8 | 1771  CLL1390  CML-10C2  17CM98  D-17  Bliley | Welch's t-test | <0.001 |
| IL-8 | Abrams  CML-6M  CMT12  D-17  Nike  Parks | Vogel  1771  OS2.4  OSA8  Yamane  STSA-1 | Mann-Whitney | 0.0022 |
| KC-like | Abrams  D-17  17CM98  Gracie  Parks  1771 | STSA-1  Moresco  Vogel  Yamane  MacKinley  CTAC | Student's  t-test | <0.001 |
| IL-10 | Nike  OSA8  CML-6M  CMT12  CML-10C2  DH82 | CLL1390  Moresco  D-17  OS2.4  Jones  MacKinley | Mann-Whitney | 0.0022 |
| CCL2 | HMPOS  Parks  OS2.4  STSA-1  Bliley  DH82 | Nike  1771  D-17  17CM98  MacKinley  OSA8 | Student's  t-test | <0.001 |
| TNF-α | OSA8  Nike  CML-6M  CMT12  DH82  Abrams | CLL1390  D-17  Moresco  CTAC  1771  Bliley | Mann-Whitney | 0.0022 |
| TGF-β | MacKinley  CMT12  Jones  Parks  Gracie  CML-6M | Abrams  17CM98  1771  CML-10C2  Bliley  Nike | Welch's  t-test | 0.0019 |

Top and bottom quartiles (n=6 each) were identified based on cytokine modified z-scores and used for pairwise gene expression analysis. "Test" indicates the statistical method used to confirm significant differences in cytokine expression between quartiles.
