# Supplementary material for: Distinct tumor genomic signatures underlie canine macrophage polarization
Source: PLoS One. 2026 Apr 24;21(4):e0346239. doi: 10.1371/journal.pone.0346239 (PMC13108725; doi:10.1371/journal.pone.0346239)
Supplement: S8 Table — Model included donor as random effect (n = 4 donors, 4 doses per donor, 16 total observations). (DOCX) [file pone.0346239.s012.docx]

**S8 Table.** Linear mixed-effects regression analysis of CCL3 dose effect on TNF-α secretion.

| Term | Estimate | SE | 95% CI | t | p.value | DF |
| --- | --- | --- | --- | --- | --- | --- |
| (Intercept) | 0.1349 | 0.0193 | [0.0923, 0.1774] | 6.98 | <0.001 | 11 |
| CCL3 dose | -0.0183 | 0.0034 | [-0.0258, -0.0108] | -5.37 | <0.001 | 11 |
| Response variable was inverse-transformed (1/TNF-α) | | | | | | |
| Model fit: AIC = -42.34 , BIC = -39.79 | | | | | | |

Model included donor as random effect (n = 4 donors, 4 doses per donor, 16 total observations).
