# Supplementary material for: Distinct tumor genomic signatures underlie canine macrophage polarization
Source: PLoS One. 2026 Apr 24;21(4):e0346239. doi: 10.1371/journal.pone.0346239 (PMC13108725; doi:10.1371/journal.pone.0346239)
Supplement: Attachment — Submitted filename: Response to Reviewers.docx [file pone.0346239.s014.docx]

**Response to Reviewers**

**Manuscript ID:** PONE-D-25-56168

**Title:** Distinct tumor genomic signatures underlie canine macrophage polarization

Dear Dr. Nie and Reviewer,

Thank you very much for the opportunity to revise our manuscript and for the very thoughtful and constructive feedback. We appreciate the reviewer's recognition of the study's contributions to comparative oncology and canine immuno-oncology. We have carefully addressed all comments and believe the revisions have substantially strengthened the manuscript.

Below, we provide point-by-point responses to each comment. Reviewer comments are shown in **bold**, and our responses follow.

# Journal Requirements

## Requirement 1: PLOS ONE Style Requirements

**Please ensure that your manuscript meets PLOS ONE's style requirements, including those for file naming.**

**Response:** We have revised the manuscript to comply with PLOS ONE style guidelines. Specific changes include: state abbreviations in affiliations spelled out in full (e.g., "Colorado" instead of "CO"); figure citations use "Fig" format; continuous line numbering implemented throughout; all abbreviations defined at first use; and Vancouver reference style verified. All figure and supporting information files have been fixed to comply with PLOS ONE conventions.

## Requirement 2: Code Sharing

**Please review our guidelines on code sharing and ensure that your code is shared in a way that follows best practice.**

**Response:** All R scripts used for statistical analysis, visualization, and RNA-sequencing analysis have been deposited in a public GitHub repository with a DOI from Zenodo.

We would like to update our Data Availability statement as follows:

"Analysis code is available on GitHub (<https://github.com/rbrady783/TCM-macrophages>) and archived on Zenodo (DOI: 10.5281/zenodo.18499208). Raw data are available on Dryad (DOI: 10.5061/dryad.brv15dvnx).”

(Raw data are available on Dryad. **Please note that the Dryad DOI (10.5061/dryad.brv15dvnx) is reserved** and will become active upon publication of the manuscript. In the meantime, the raw data can be accessed via Dryad's pre-publication review link: <http://datadryad.org/share/LINK_NOT_FOR_PUBLICATION/4TdabHt6t8I-dNvhnq9Mp3wjyTFsqiWv2u_H1s9MUvE>).

**Requirement 3: Role of Funders**

**Please state what role the funders took in the study.**

**Response:** The following statement is correct: "The funders had no role in study design, data collection and analysis, decision to publish, or preparation of the manuscript."

## Requirement 4: Data Availability

**We strongly recommend all authors decide on a data sharing plan before acceptance.**

**Response:** All data underlying the findings are now available. See above for DOIs. **Please note that the Dryad DOI (10.5061/dryad.brv15dvnx) is reserved** and will become active upon publication of the manuscript. In the meantime, the raw data can be accessed via Dryad's pre-publication review link: <http://datadryad.org/share/LINK_NOT_FOR_PUBLICATION/4TdabHt6t8I-dNvhnq9Mp3wjyTFsqiWv2u_H1s9MUvE>).

## Requirement 5: Blot/Gel Images

**Please provide the original uncropped and unadjusted images underlying all blot or gel results.**

**Response:** Original uncropped western blot images for CCL3 knockdown validation are provided in S1_raw_images. These images show the full membrane for both CCL3 and the α-tubulin loading control, with lanes clearly labeled.

## Requirement 6: "Unpublished Data" References

**PLOS does not permit references to inaccessible data.**

**Response:** The reference to unpublished data regarding CCL3 expression in canine histiocytic sarcoma has been revised. We now only cite publicly available RNA-seq datasets (NCBI BioProjects PRJDB11462, PRJEB36828, PRJDB17594) that support the finding that CCL3 is broadly expressed in canine HS tumors and cell lines. The personal communication reference has also been removed.

# Reviewer #1 Comments

We thank Reviewer #1 for the thoughtful and expert evaluation of our work. The reviewer's comments have significantly improved the manuscript's focus and impact.

## Major Comments

### Major Comment 1: Introduction is overly human-centric

**"While the Introduction is comprehensive, the majority of cited literature and conceptual framing is centered on human cancer biology... The Introduction should be re-structured to center companion animals earlier and more prominently."**

**Response:** We agree that the Introduction was imbalanced. We have substantially revised it to: (1) center canine cancer biology earlier, establishing companion animal oncology as the primary focus; (2) expand discussion of knowledge gaps in canine tumor immunology, including the paucity of validated biomarkers and limited mechanistic understanding of macrophage-tumor crosstalk in dogs; (3) position canine cancer as biologically informative in its own right, not merely a translational bridge; and (4) reduce generic human TME content.

### Major Comment 2: TME section disproportionately long

**"The general TME overview is overly extensive, whereas the section on canine immunotherapy and TAM biology is comparatively brief."**

**Response:** We have rebalanced the Introduction by substantially condensing the generic TME discussion (heterogeneity, metabolic reprogramming, epigenetic regulation) and expanding canine-specific content, including known gaps in canine immunotherapy development, limited availability of validated immune biomarkers in veterinary oncology, and specific challenges in translating TAM-targeted therapies to companion animals.

### Major Comment 3: "Biomarker" used too broadly

**"The term biomarker is used somewhat loosely... substantial additional work would be required before these could function as predictive or clinical biomarkers."**

**Response:** We agree and have revised terminology when referring to our own findings, using 'candidate biomarker,' 'potential biomarker,' or similar qualifiers. We have added explicit acknowledgment in the Discussion that significant additional validation is required before these correlates could serve as predictive or therapeutic biomarkers. We have also slightly expanded some of our limitations to more fully explain the weaknesses of our experimental system.

### Major Comment 4: Exosome findings under-emphasized

**"The exosome findings represent the most innovative and distinguishing aspect of the study... I strongly recommend elevating the exosome story more prominently."**

**Response:** We thank the reviewer for this important suggestion. We have: (1) revised the Abstract to more prominently feature the exosome findings; (2) moved the exosome Discussion section earlier and expanded it to highlight how this mechanism may differ from human systems and to frame exosome-mediated macrophage reprogramming as a key feature of canine tumor immunology; (3) added a more prominent Results subheading; and (4) highlighted the exosome finding as a primary contribution in the Conclusions.

### Major Comment 5: Manuscript structure and figure placement

**"The current structure, with long figure legends interspersed throughout the Results, makes the manuscript difficult to read."**

**Response:** We appreciate this feedback. We understood PLOS ONE submission guidelines to require figure legends be included within the manuscript text rather than as separate captions attached to figures. We have placed boxes around figure legends to help visually distinguish them from the main text. We have also tried to improve descriptive subsection headers throughout Results to guide the reader through the narrative and ensure conceptual advances are described in the text before directing readers to figures. We have shortened some of our Figure legends as well.

## Minor and Technical Comments

### Minor Comment 1: Supplementary tables

**"Supplementary tables are frequently referenced, but their contents are not always sufficiently described in the main text."**

**Response:** We have added brief in-text descriptions for each supplementary table when first referenced, including the variables included, sample sizes, and analytical purpose.

### Minor Comment 2: R scripts and reproducibility

**"It is unclear whether these scripts are available and where readers can access them."**

**Response:** All R scripts have been deposited in a public GitHub repository with an archived DOI via Zenodo. The repository includes data processing scripts, statistical analysis code, DESeq2 pipeline, GSEA scripts, and figure generation code. The repository URL and Zenodo DOI have been added to the Data Availability Statement.

### Minor Comment 3: Pathway analysis choices (KEGG)

**"It would be helpful to explain why KEGG libraries were not included."**

**Response:** We have added a brief justification in the Methods: " KEGG pathway analysis was not included as MSigDB's C2 collection incorporates KEGG-derived gene sets alongside other curated sources."

### Minor Comment 4: "Cell counts at TCM harvest" clarification

**"The phrase 'cell counts at TCM harvest' is confusing... It is unclear whether this refers to tumor cells, macrophages, or another population."**

**Response:** We have clarified this throughout the manuscript. The phrase now reads 'tumor cell count at TCM harvest' to unambiguously refer to cancer cell counts.

### Minor Comment 5: Canine-specific immunology emphasis

**"Several Discussion sections revert to human analogies... The manuscript would benefit from more explicit statements highlighting what we still do not understand about canine immune regulation."**

**Response:** We have revised the Discussion to lead with canine-specific findings before drawing human parallels, add explicit statements about gaps in canine knowledge, and frame comparative insights as bidirectional i.e. what canine studies can teach us about human biology.

# Additional Revisions

In addition to addressing reviewer comments, we made the following clarifications:

**CCL3 knockdown quantification:** We revised the description of CCL3 knockdown efficiency to use consistent percentage language. The original text stated “71% reduction vs NTC (55% vs WT),” which mixed reduction terminology with residual expression and contained an arithmetic error in the NTC comparison. Based on the raw densitometry values (normalized to α-tubulin loading control), this has been corrected to “45% of WT levels (61% of NTC)” to match the “% of” phrasing used elsewhere in the manuscript (e.g., ELISA results).

**Excluded analytes moved to Results:** The sentence describing eight analytes excluded due to measurements below the lower limit of quantification (LLoQ) has been moved from Methods (Cytokine analysis) to Results, as this was a data-driven finding rather than a pre-specified methodological decision.

# Closing

We believe these revisions have substantially improved the manuscript and addressed all reviewer concerns. We thank the editor and reviewer for their time and constructive feedback.

Very Sincerely,

Rachel V. Brady, DVM, PhD

On behalf of all authors
